# Metabolites in Milk after Enrofloxacin Treatment and Their Persistence to Temperature

**DOI:** 10.1021/acs.jafc.2c02230

**Published:** 2022-07-01

**Authors:** Alexandra Junza, Javier Saurina, Cristina Minguillón, Dolores Barrón

**Affiliations:** † Department de Enginyeria Química i Química Analí́tica, Universitat de Barcelona, Martí i Franquès, 1-11, Barcelona 08028, Spain; ‡ Department de Nutrició, Ciències de l’alimentació i Gastronomia, Universitat de Barcelona, Avda. Prat de la Riba, 171, Sta. Coloma de Gramenet, Barcelona 08921, Spain; § Institut de Recerca en Nutrició i Seguretat Alimentària de la Universitat de Barcelona (INSA-UB), Barcelona 08007, Spain

**Keywords:** cow milk, biomarkers, enrofloxacin, metabolomic profile, metabolites

## Abstract

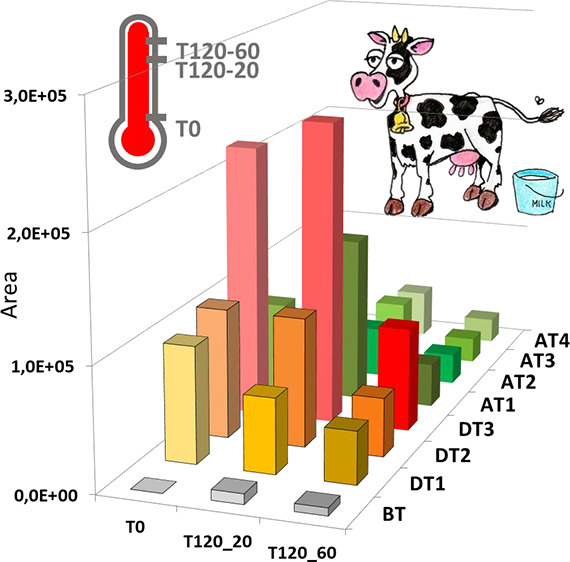

In this work, metabolomic profile changes in milk from cows affected by mastitis and treated with enrofloxacin (ENR) have been studied using LC-HRMS techniques. Principal component analysis was applied to the obtained results, and the interest was focused on changes affecting compounds without a structural relationship to ENR. Most of the compounds, whose concentrations were modified as a result of the pharmacological treatment and/or the pathological status, were related to amino acids and peptides. Compounds that may become possible biomarkers for either disease or treatment have been detected. Additionally, the alterations caused by thermal processes, such as those applied to milk before consumption, on the identified metabolites have also been considered.

## 1 Introduction

Mastitis, either clinical or subclinical, is the infection with the highest prevalence among dairy cows. It affects the yield of produced milk as well as its quality. Mastitis is mostly caused by bacteria and it is treated in farms with the administration of antibacterial agents such as quinolones, β-lactam antibiotics, and macrolide and/or aminoglycoside antibiotics.^1−3^

Several studies have demonstrated modifications in the amount and kind of endogenous low-molecular-weight metabolite in animals suffering from mastitis. Therefore, the metabolomic profile of the milk produced is also modified.^4−6^ Additionally, the pharmacological treatment with antibacterial agents also contributes to such changes in the metabolome. For instance, the abuse or improper use of antibiotics may lead to residues in milk, especially when withdrawal times are not fulfilled.^7^

To ensure the safety of milk, the European Union (EU) authorities have established maximum residue limits (MRLs) for veterinary drugs in food of animal origin intended for human consumption.^8^ However, only the administered drug is considered and no attention is given to metabolites and/or derivatives produced because of the pharmacological treatment applied.

Moreover, in the dairy industry, milk is subjected to diverse thermal processes before being marketed. The most frequently applied are flash pasteurization (HTST) (72 °C, 15 s, high-temperature/short-time treatment), sterilization (120 °C, 20 min, high-temperature/long-time treatment), and UHT sterilization (140 °C, 4 s, ultrahigh-temperature/short-time treatment). Such processes are addressed to ensure milk safety and preservation by destroying the possible pathogens that may be present. Regarding milk and its thermal treatment before consumption, a recent work has undertaken the metabolomic discrimination between UHT and reconstituted milk.^9^ Also, pasteurized milk has been the object of another study in which the search for biomarkers to predict the shelf-life of the milk subjected to such treatment was the aim.^10^

Although milk is a very heat-stable system in comparison to other food materials, the applied treatment affects not only microorganisms but also milk components. For instance, sterilization is known to produce some browning resulting from Maillard reactions and the loss of lysine and certain vitamins. Nevertheless, scientific data about the effect that milk-processing temperatures/treatments may have in the presence of antimicrobial residues are scarce.

The stability of various quinolones during storage at different temperatures and after processes similar to those applied to milk before consumption was studied.^11−13^ Quinolones were determined to be very resistant to different heat treatments.^11^ Estimated maximum decreases in concentration of around 12% for ciprofloxacin and norfloxacin after heating at 120 °C during 20 min were determined. However, this value is even lower for other quinolones, which implies that these antibacterial agents may reach consumers.

In addition, depending on the temperature and time involved in the subsequent thermal treatment, antibiotic residues and metabolites undergo diverse transformations.^14,15^ As the products formed could be even more persistent than the drugs administered, the emergence of unexpected metabolites, or the observation of an altered metabolomic profile, may constitute biomarkers of pathologic state and/or pharmacological treatments, which would be of interest in the evaluation of milk quality and safety.^4^

Sensitive analytical methodologies are needed to monitor the possible occurrence of residues, metabolites, or transformation products, which are present at very low concentrations in food products. LC-HRMS techniques (LC-TOF and LTQ-Orbitrap MS) are the most adequate to simultaneously accomplish the detection/quantification and elucidation of structures of compounds formed during the thermal treatment of milk.^16−18^

In this context, the aim of this work is to locate metabolomic modifications in cow milk detectable after the administration of enrofloxacin (ENR) and to study their fate after a thermal treatment. Without the pretension of being exhaustive and as a continuation of preceding studies,^14,15^ focusing mainly on ENR-related metabolites, the spotlight of this study is on those compounds not structurally related to the antibacterial agent. The detected compounds may become possible biomarkers for treatment or disease.

## 2 Materials and Methods

### 2.1 Chemicals and Reagents

Methanol (MeOH) and acetonitrile (MeCN) of analytical grade were purchased from Panreac (Castellar del Vallès, Spain). Formic acid (HCOOH), sodium dihydrogenphosphate (NaH_2_PO_4_), and sodium hydroxide (NaOH), all of analytical grade, were supplied by Merck (Darmstadt, Germany). Water (18.2 MΩ·cm) was generated using a Milli-Q purification system from Millipore (Billerica, MA, USA).

The buffer solution of sodium dihydrogenphosphate (NaH_2_PO_4_; 0.1 mol·L^–1^) used in the treatment of samples was adjusted to pH 10 with 5 mol·L^–1^ NaOH. Oasis HLB polymeric cartridges (3 cm^3^, 60 mg) from Waters (Milford, MA, USA) were used for solid-phase extraction. Membrane filters (Ultra free Durapore PVDF 0.22 μm) from Millipore were used to filter the extract before analysis.

### 2.2 Sample Collection

Milk samples were provided by the farm La Saireta S.C.P. (Vallfogona de Balaguer, Lleida, Spain). Medication consisted of an intramuscular administration of 5 mg·kg^–1^/day Enrovet (Group Divasa Farmavic, Vic, Spain), in which the active ingredient is ENR. The pharmacological treatment lasted for 3 days. Milk samples were collected before treatment (BT), 3 days of drug administration (DT1–DT3), and 4 days immediately after treatment (AT1–AT4). Samples were stored at −20 °C prior to analysis.

### 2.3 Heat Treatment of Samples

Samples DT1–DT3 and AT1–AT4 were prepared in triplicate and BT in quadruplicate. To study the thermal stability of metabolites, cow milk samples were heated at 120 °C for either 20 or 60 min (samples referred to as T120.20 and T120.60, respectively) before analysis. Control samples (T0) were not subjected to the heating process.

### 2.4 Sample Preparation

Sample processing consisted of a method already described.^15^ Thus, milk samples, previously diluted with Milli-Q water and NaH_2_PO_4_ (0.1 mol·L^–1^) solution at pH 10, were subjected to solid-phase extraction. The eluted fractions were evaporated to dryness, and the extracts were reconstituted with 200 μL of Milli-Q water and filtered before analysis.

### 2.5 Chromatographic and Mass Spectrometry Conditions

Samples were analyzed using an HP Agilent Technologies 1100 LC system coupled to a 6220 ToF LC/MS mass spectrometer. Ionization was performed by an ESI source (Agilent Technologies, Santa Clara, CA, USA) working in positive mode.

Chromatographic separation was performed using a Zorbax Eclipse XDB-C8 analytical column (150 × 4.6 mm) with 5 μm particle size (Agilent Technologies) equipped with a Kromasil C8 (4.6 × 15 mm, 5 μm) precolumn supplied by Akady (Barcelona, Spain) and eluted at a 1 mL·min^–1^ flow rate. The mobile phase consisted of the following: A, water with 0.1% HCOOH; B, MeCN with 0.1% HCOOH. The gradient was programmed as follows: initial 15% B for 1 min; from 1 to 4 min, B increased to 45%; from 4 to 7 min, B increased to 56%; from 7 to 8.5 min, B decreased to 15%; B was maintained until 11 min. The injection volume was 20 μL. The main parameters of ToF-MS were as follows: capillary voltage, 4000 V; drying gas (N_2_) temperature, 300 °C; drying gas (N_2_) flow rate, 9 L·min^–1^; nebulizer gas (N_2_), 40 psi; fragmentor voltage, 150 V; skimmer voltage, 60 V; OCT 1 RF voltage, 250 V. The spectra were acquired over the *m*/*z* 50–1100 range. The mass resolving power was approximately 10,000 FWHM at *m*/*z* 922. Data storage was in profile and centroid modes.

The data used in the elucidation of metabolites and transformation products were obtained using an Accela LC system coupled to an LTQ Orbitrap Velos mass spectrometer with an ESI source in positive mode (Thermo Scientific, Hemel Hempstead, UK). The column used during the chromatographic analysis of samples was a Pursuit UPS C18 column (50 × 2.1 mm) with 2.4 μm particle size (Varian, Harbor City, CA, USA). Mobile phase solutions A and B have the same composition as those described above. The chromatographic method applied was as follows: initial 1% B for 3.5 min; from 3.5 to 4.5 min, B increased to 25%; from 4.5 to 5 min, B increased to 50%; from 5 to 6.5 min, B was maintained at 50%; from 6.5 to 7.5 min, B decreased to 25%; from 7.5 to 8.5 min, B decreased to initial conditions and was maintained until 11 min. The mass range was set from *m*/*z* 100 to 700. Milk samples were first analyzed in full MS mode using the Orbitrap system in which the mass resolving power was set at 30,000 FWHM at *m*/*z* 400. The MS^*n*^ analyses were performed with the same system having the resolving power set at 15,000 FWHM at *m*/*z* 400. The optimized conditions were as follows: source voltage, 3.5 kV; sheath gas (N_2_), 40 (arbitrary units); auxiliary gas (N_2_), 10 (arbitrary units); sweep gas (N_2_), 10 (arbitrary units); capillary temperature, 275 °C. Default values were used for most other acquisition parameters (Fourier transform (FT); automatic gain control (AGC) target values of 1 × 10^6^ for MS mode and 5 × 10^4^ for MS^*n*^ mode). The maximum injection times were set at 100 ms with one microscan for MS mode and at 500 ms with one microscan for MS^*n*^ mode.

### 2.6 Auxiliary Equipment

Samples were heated in a laboratory oven (Selecta, Barcelona, Spain). A VX-200 vortex mixer from Labnet International (Edison, NJ, USA) and a Rotanta 460RS centrifuge from Hettich Zentrifuguen (Tuttlingen, Germany) were also used for sample treatment. A Supelco vacuum manifold with disposable liners for 24 cartridges connected to a Supelco vacuum tank (Bellefonte, PA, USA) was used in the SPE step. Extract evaporation was carried out into a miVac Duo Concentrator of Genevac (Ipswich, England). A Crison 2002 potentiometer (± 0.1mV) with a Crison 5203 combined pH electrode (Barcelona, Spain) was used to adjust the pH of the phosphate buffer solution.

### 2.7 Data Treatment

Data obtained from the analysis by LC-ToF were treated using the MZmine 2 free software.^19^ A previous format change of data file (from .d to an open .mzXML) was performed with Trapper.^20^

Data extracted by MZmine, considering a tolerance in a retention time of 10 s and in *m*/*z* of 5 ppm, were composed of 2021 ion features for the set under study. Such a matrix contained some ion artifacts associated with the dead volume and cleaning range of the chromatogram. Thus, it was filtered considering the following restrictions: working time range (*t*_R_ = 2–8.5 min), mass range (*m*/*z* 200–550), and a mass defect filter (0.04 < MDF < 0.24). A list of *m*/*z* ratios for each sample (BT, DT1–DT3, and AT1–AT4) and each heating treatment (T0, T120.20, and T120.60) was obtained accordingly, with a dimension of 75 samples × 895 features, which was subjected to a preliminary exploratory study by principal component analysis (see below).

Further filters were applied to obtain a reduced matrix to facilitate the identification of the most relevant discriminant features and improve the robustness of the description. Concretely, only those ions displaying mean intensity values higher than 10,000 in samples (BT, DT, and AT) were retained as potential markers of BT, DT, and AT classes. An additional RSD filter, based on the calculation of the precision for ion intensities of each sample triplicate, was applied. Those ions with mean RSD percentages lower than 20% were taken for analysis, while those displaying higher variability were excluded. Under these circumstances, the resulting matrix was 75 samples × 149 variables.

### 2.8 Principal Component Analysis

SOLO software^21^ (Eigenvector Research, Manson, WA) was used in principal component analysis (PCA) and related methods. A detailed description of the theoretical background of these methods is given elsewhere.^22^

In all instances, data were autoscaled to provide similar weights for all features. The score plot, showing the distribution of samples based on principal components (PCs), was used to reveal patterns for sample characteristics such as day collected and thermal process applied. The loading plot showed the distribution of variables on the space of PCs to gain information dealing with ions and their correlations.

## 3 Results and Discussion

The search for possible metabolomic modifications in milk started with the comparison of the list of *m*/*z* values obtained for each milk sample collected during (DT) or after treatment (AT) with that obtained from samples collected before treatment (BT) for all three thermal conditions. Those ions accomplishing certain fundamental requirements were included in the final list of compounds deserving further study. Therefore, compounds appearing in samples DT1–DT3 and AT1–AT4, but not in BT, were considered. Compounds present in milk because of the illness evolution and after establishing the treatment may be found in this group. Compounds whose amounts decreased under the detection limit, either because of the illness or as a consequence of the installation of the treatment, would be in this group. Finally, those compounds appearing in the two, before (BT) and after establishing the treatment (DT and/or AT), whose area ratios have been significantly modified by this fact, were also taken into account. Thus, ions showing an area ratio of XT/BT (being XT either DT or AT) or BT/XT higher than 10, which was increased or decreased with pathology/treatment, respectively, were considered. The procedure was applied to nonthermally treated samples (T0) as well as heated samples (T120.20 and T120.60). To avoid artifacts, attention has been given to the presence of the same *m*/*z* value at least in two consecutive days. The above-described procedure permits one to select 149 ions in BT, DT1–DT3, and/or AT1–AT4 samples in the positive ionization mode. A full list of compounds ordered by increasing *m*/*z* exact mass is provided in the Supporting Information (Table A). The numeration of compounds is maintained all throughout this study.

### 3.1 PCA

A preliminary PCA model was established using a raw data matrix (72 × 895) as described in Section 2. In this model, contributions from the dead volume front and the cleaning ranges of the chromatogram were removed. Results are included in the Supporting Information (Figure A). It should be remarked that these results rely on a model without any assumption about the nature of the potential markers/descriptors of the process. The score plot shows clear sample discrimination depending on these two factors. In this preliminary study, PC1 mainly describes the influence of the illness/drug treatment on the sample distribution, with the samples before treatment (BT) on the left side, those collected during the drug administration (DT1 to DT3) in the central part, and the post-treatment samples (AT1 to AT4) on the bottom-right area. Unfortunately, it is not possible to attribute changes in the metabolomic fingerprints exclusively to the drug treatment or to the evolution of the illness itself. The influence of the thermal treatment is mainly modeled by PC2. It should be noted that, in any case, samples untreated thermally tend to be on the top of others of similar characteristics, while those subjected to 120 °C for 60 min (i.e., the most intense thermal treatment) are located on the bottom part.

**Figure 1:**
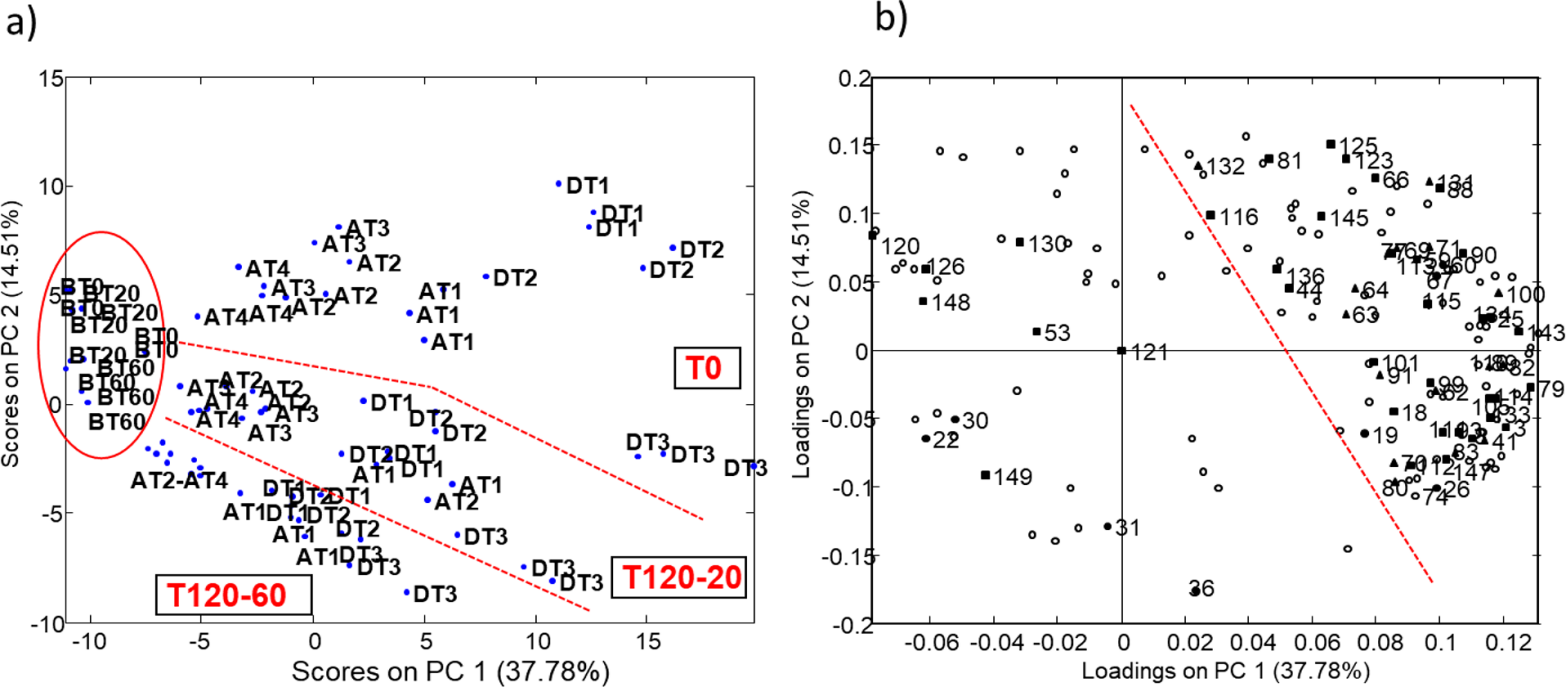
(a) PCA score plot of PC1 *vs* PC2 showing the grouping of milk samples for pharmacological and thermal treatments. (b) Plot of loadings showing the distribution of variables on PC1 and PC2. Previously identified, solid up-pointing triangle; database METLIN, solid square; identified in this work, solid circle; not identified, empty circle.

After this preliminary exploration, the filtered data matrix (75 × 149) was analyzed by PCA. The study of scatter plots of PC1 *vs* PC2 (Figure 1a) provides significant information on the clustering of the diverse samples that maintain the same trends as the preliminary study.

Components PC1 and PC2 displayed 37.8 and 14.5% of data variance, respectively. Samples from nontreated cows (BT) appear in the score plot as a compact group on the left of the graph. This is not the case for milk samples collected during or after medication (DT and AT). Among them, those that are not subjected to thermal treatment are mainly located at the top of the graph, while those heated for 20 min (T120.20) appear below them and those heated for 60 min (T120.60) are spread at the bottom-left corner of the graph. This dispersion may be an indication of the thermal sensitivity of the compounds present in DT and AT samples. As for the milking time, samples collected during the veterinary treatment (DT1 to DT3) are located to the right side of the graph, while those corresponding to the post-treatment period (AT1 to AT4) tend to be closer to BT samples at the left side of the graph. Therefore, when the collection day is considered, samples follow a similar pattern regardless of the thermal treatment. Thus, the sample DT3 is located to the right of the graph, while AT2 to AT4 tend to be to the left side.

In summary, it can be considered that the PC1 component mainly describes the sample behavior as a function of drug and altered metabolite content. Samples with the expected highest concentration of drug and altered metabolites are on the right side of the graph, while samples without drug (i.e., BT) and with a predictable low level of drug residues (i.e., those collected at the end of the post-treatment period such as AT4) are on the left. In addition, the PC2 component mainly explains the effect of the thermal treatment on the compositional profiles of milk samples. Clearly, unheated samples are at the top of this graph, while those subjected to the most intense treatment tend to be located at the bottom. The trends observed for the reduced set of compounds are the same as those observed for a previous set including 895 compounds (Figure A, Supporting Information). This fact constitutes an indication of the validity of the stated conclusions to this respect.

The loading plot (Figure 1b) shows the distribution of variables on PC1 and PC2. Most ions are located at the right side of the plot. Compounds such as ENR (**100**), CIP (**80**, ciprofloxacin), and other drug metabolites already detected in previous studies are located in this area. Nevertheless, a significant number of compounds are distributed throughout the graph.

### 3.2 Determination and Characterization of Selected Metabolites

At this point, the aim was to identify as many compounds on the list as possible. In the first approach, compounds already described in precedent studies, most of them related to the ENR structure, were located on the list (19 compounds out of 149).^14,15,23^ Compounds such as **41**, **69**, **78**, and **80** (ciprofloxacin), among others, are structurally related to ENR (**100**) and its conjugates with amino acids, i.e., metabolites of phases I and II of ENR (Table 1).

**Table 1:** Experimental [M + H]^+^, Retention Time, Molecular Formula, Error, and Tentative Identification of the Compounds Studied

compound number	[M + H]^+^_exp_	RT (min)	molecular formula	error (ppm)	tentative identification	source
**3**	204,1356	2.2	C_8_H_18_N_3_O_3_^+^	6.4	Lys/Gly	(25)
**8**	215,1405	3.9	C_10_H_19_N_2_O_3_^+^	7.0	Pro/Val	(25)
**18**	231,1702	2.3	C_11_H_23_N_2_O_3_^+^	6.1	Val/Leu, Val/Ile	(25)
**19**	231,1702	3.0	C_11_H_23_N_2_O_3_^+^	2.9		a
**22**	239,1375	7.7	C_12_H_19_N_2_O_3_^+^	4.6		a
**25**	245,1871	3.4	C_12_H_25_N_2_O_3_^+^	4.5	Ile-Ile, Leu/Ile	(25)
**26**	245,1871	5.1	C_12_H_25_N_2_O_3_^+^	2.9		a
**30**	249,1859	3.9	C_16_H_25_O_2_^+^	4.0		a
**31**	251,1511	2.1	C_12_H_20_N_4_O_2_^+^	3.2		a
**36**	256,1777	3.2	C_11_H_23_N_5_O_2_^+^	3.5		a
**41**	263,0844	6.9	C_13_H_12_FN_2_O_3_+	3.0	ENR-1	(15,23)
**44**	265,1560	2.1	C_14_H_21_N_2_O_3_^+^	4.9	Phe/Val	(25)
**53**	287,1159	5.7	C_11_H_19_N_4_O_3_S^+^	–4.5	His/Met	(25)
**59**	289,1803	3.2	C_18_H_25_O_3_^+^	1.7	hydroxyestradiol	(25)
**60**	295,1666	3.7	C_15_H_23_N_2_O_4_^+^	4.7		a
**62**	302,1348	3.4	C_12_H_20_N_3_O_6_^+^	0.3	Pro/Gly/Glu	(14)
**63**	302,1348	4.1	C_12_H_20_N_3_O_6_^+^	0.3	Pro/Gly/Glu	(14)
**64**	302,1348	4.5	C_12_H_20_N_3_O_6_^+^	0.3	Pro/Gly/Glu	(14)
**65**	302,1348	5.7	C_12_H_20_N_3_O_6_^+^	0.3	Pro/Gly/Glu	(14)
**66**	304,1312	4.5	C_12_H_22_N_3_O_4_S^+^	–4.6	Pro/Met/Gly	(25)
**67**	304,2130	4.0	C_15_H_30_NO_5_^+^	4.0		a
**69**	306,1256	3.6	C_15_H_17_FN_3_O_3_^+^	2.6	ENR-3	(15)
**70**	306,1281	2.3	C_11_H_20_N_3_O_7_^+^	–4.9	Ala/Asp/Thr, Thr/Glu/Gly	(25)
**71**	307,1242	2.5	C_10_H_19_N_4_O_7_^+^	–2.0	Ser/Ans/Ser	(25)
**77**	329,1498	3.5	C_16_H_25_O_7_^+^	0.6	PheTyr	(14)
**78**	329,1498	5.0	C_16_H_25_O_7_^+^	0.6	compound **15**	(14)
**79**	330,1650	4.2	C_14_H_24_N_3_O_6_^+^	–3.0	Asp/Pro/Val	(25)
**80**	332,1408	5.1	C_17_H_19_FN_3_O_3_^+^	0.9	ciprofloxacin	(14,15)
**81**	332,1826	8.4	C_14_H_26_N_3_O_6_^+^	3.0	Glu/Ala/Leu, Val/Val/Asp	(24,25)
**82**	334,1207	6.4	C_16_H_17_FN_3_O_4_^+^	2.7	ENR-5	(15)
**83**	334,1566	5.0	C_17_H_21_FN_3_O_3_^+^	1.5	ENR-6	(15)
**84**	334,1775	5.8	C_17_H_24_N_3_O_4_^+^	1.7		a
**88**	345,2141	2.2	C_15_H_29_N_4_O_5_^+^	2.6	Ile/Val/Asn	(25)
**89**	346,1204	6.5	C_17_H_17_FN_3_O_4_^+^	1.7	ENR-7	(15)
**91**	350,1705	4.8	C_17_H_24_N_3_O_5_^+^	1.4	compound **16**	(14)
**93**	352,1849	2.5	C_17_H_26_N_3_O_5_^+^	–5.1	Leu/Tyr/Gly	(25)
**99**	360,1357	6.9	C_18_H_19_FN_3_O_4_^+^	0.8	ENR-10	(15,23)
**100**	360,1721	5.7	C_19_H_23_FN_3_O_3_^+^	0.8	enrofloxacin	(14,15)
**101**	360,1953	2.5	C_16_H_30_N_3_O_4_S^+^	0.3	Pro/Leu/Met	(25)
**105**	366,2035	2.8	C_18_H_28_N_3_O_5_^+^	3.3	Phe/Ser/Leu	(25)
**111**	373,1694	5.1	C_15_H_25_N_4_O_7_^+^	–6.4	Glu/Pro/Gln	(25)
**112**	373,2443	2.5	C_17_H_33_N_4_O_5_^+^	–0.5	Leu/Leu/Gln	(24)
**113**	376,1705	4.5	C_19_H_23_FN_3_O_4_^+^	1.1	ENR-16	(23)
**114**	376,1925	3.6	C_13_H_26_N_7_O_6_^+^	–3.7	Arg/Asn/Ser	(25)
**115**	376,2163	5.5	C_15_H_30_N_5_O_6_^+^	–7.4	Lys/Thr/Gln	(25)
**120**	385,2205	6.7	C_16_H_29_N_6_O_5_^+^	2.9	Thr/His/Lys	(25)
**121**	387,2023	7.0	C_15_H_27_N_6_O_6_^+^	9.3	Pro/Asp/Arg	(25)
**123**	391,1730	4.2	C_22_H_23_N_4_O_3_^+^	–8.9	TrpTrp	(25)
**125**	397,1901	2.2	C_18_H_29_N_4_O_4_S^+^	–0.8	Cys/Lys/Phe	(25)
**128**	400,2050	6.1	C_17_H_30_N_5_O_4_S^+^	9.2	His/Met/leu	(25)
**130**	404,2133	6.3	C_16_H_30_N_5_O_7_^+^	–1.7	Gln/Lys/Glu	(25)
**131**	405,2135	3.4	C_20_H_29_N_4_O_5_^+^	0.7	compound **20** and isomer	(14)
**132**	405,2135	4.2	C_20_H_29_N_4_O_5_^+^	0.7	compound **20** and isomer	(14)
**133**	406,2219	2.8	C_15_H_32_N_7_O_4_S^+^	–3.0	Lys/Arg/Cys	(25)
**134**	406,2219	3.3	C_18_H_28_N_7_O_4_^+^	5.4	His/Leu/His	(25)
**136**	408,1637	2.2	C_16_H_22_N_7_O_6_^+^	2.7	His/Asp/His	(25)
**138**	412,2247	6.6	C_23_H_30_N_3_O_4_^+^	3.9	Val/Phe/Phe	(25)
**139**	419,2263	5.5	C_21_H_31_N_4_O_5_^+^	–6.2	Trp/Thr/Leu	(25)
**143**	426,2078	5.2	C_20_H_32_N_3_O_5_S^+^	4.9	Tyr/Leu/Met	(25)
**148**	434,2384	7.5	C_21_H_32_N_5_O_5_^+^	–3.2	Trp/Thr/Lys	(25)

a Elucidated in this paper.

In the next step, the literature on the subject was searched.^6,15,24^ Coincidences with compounds on the list, in terms of exact mass and significant MS fragments, were found. This allowed us to tentatively assign the structure for 34 compounds on the list (Table 1), with most of them corresponding to di- and tripeptides. An increase in the concentration of di-, tri-, and tetrapeptides in milk has been described to occur during the pathological process.^5^ This is in good agreement with the results presented here.

Finally, the identification of other compounds on the list was attempted based on their MS spectra. In Table 2, a summary of the ions tentatively elucidated in this study is presented. The proposed structure and its most relevant mass spectral data are indicated for each compound. All of them are attributed to milk components whose concentration has been altered by the pathological state, the pharmacological treatment, or the thermal process. Their structure has been proposed in most cases thanks to the comparison of fragments for other structurally related molecules in the METLIN database.

**Table 2:**
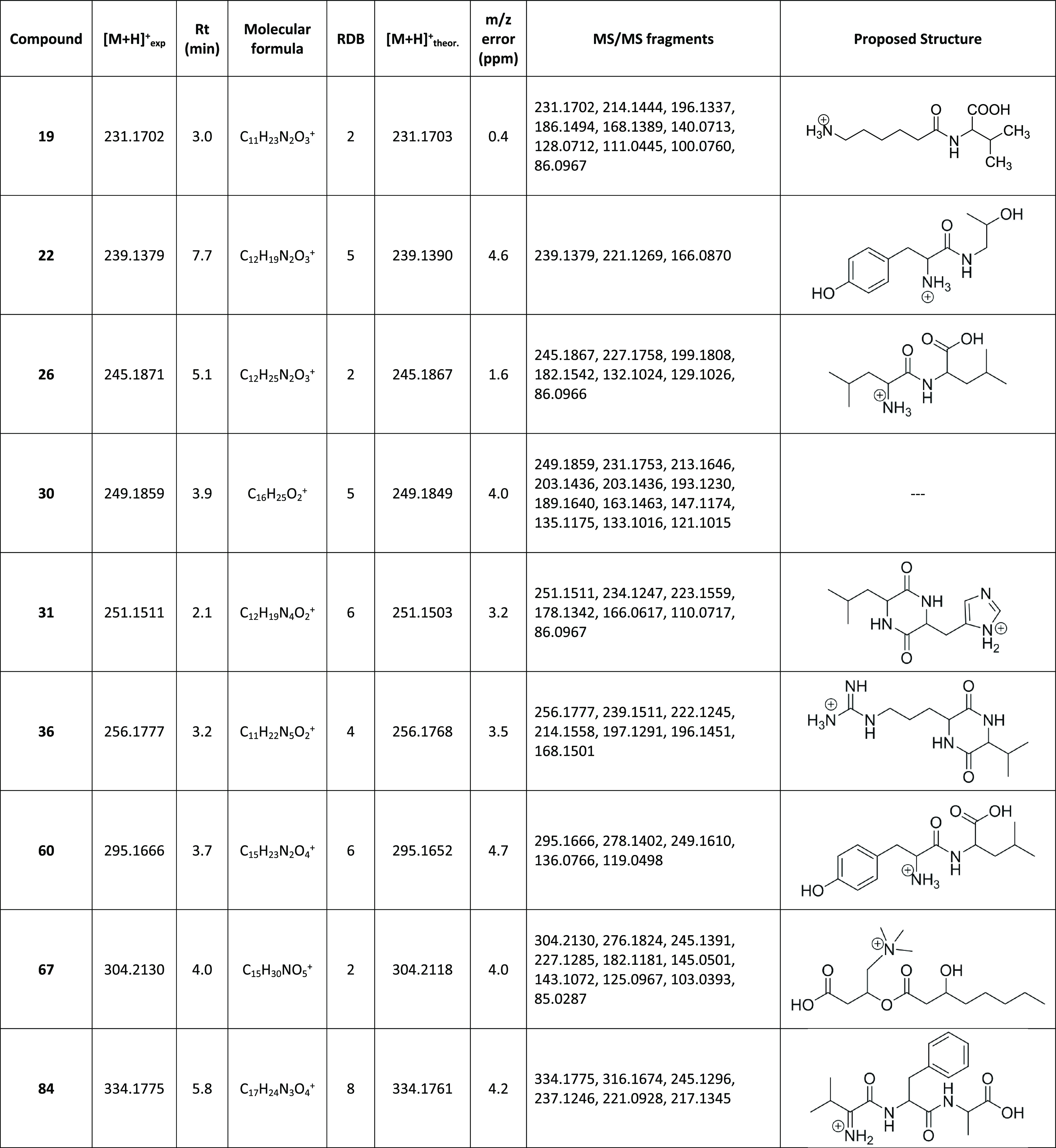
Mass Spectral Data and MS/MS Spectrum of the Identified Metabolites in Milk

The most numerous group of metabolites studied is peptides or peptide derivatives; among them are the dipeptides Leu-Leu (**26**) and Tyr-Leu (**60**) and the tripeptide Val-Phe-Ala (**84**). In the case of the dipeptide Leu-Leu (*m*/*z* 245.1871, C_12_H_25_N_2_O_3_^+^, **26**), the fragments with higher intensities, *m*/*z* 227.1758, 199.1808, and 86.0966 (Figure B in the Supporting Information), are in agreement with the fragments described for the amino acid MS/MS spectra.^25^ Additionally, the fragment *m*/*z* 132.1024 corresponds to Leu. The Tyr-Leu dipeptide (*m*/*z* 295.1666, C_15_H_23_N_2_O_4_^+^, **60**) (Supporting Information Figure C) presents main peaks at *m*/*z* 278.1402, corresponding to the loss of ammonia, and *m*/*z* 249.1610, coming from the decarboxylation of the molecular ion. The dipeptides **60** and **26** were mentioned in the study of Mansor,^24^ with the dipeptide Leu-Leu (**26**) suggested as a biomarker for mastitis. Analogously, the tripeptide Val-Phe-Ala (*m*/*z* 334.1775, C_17_H_24_N_3_O_4_^+^, **84**) presents a base peak at *m*/*z* 245.1246, which corresponds to the loss of Ala from the molecular ion. The loss of CO from this base peak is also significant, which seems to point to a diketopiperazine structure for the fragment (Supporting Information Figure D).

Compounds tentatively attributed to modified dipeptides were also identified. In this group, *m*/*z* 231.1702 (C_11_H_23_N_2_O_3_^+^, **19**) is attributed to a metabolite coming from the Val-Lys dipeptide, which has been deaminated (Supporting Information Figure E). The presence of an *m*/*z* 86.0967 peak is a characteristic of the peptides containing Val. A compound with the same *m*/*z* as **19** has been previously detected in liver tissues^26^ from chicken medicated with ENR. In that work, the low signal obtained did not allow us to perform any elucidation.

A product clearly resulting from the thermal treatment was also elucidated. The compound had *m*/*z* 239.1379 (**22**), corresponding to a molecular formula of C_12_H_19_N_2_O_3_^+^ and 5 RDB (Supporting Information Figure F), and was detected preferably in samples treated at high temperatures during a long period. The fragment *m*/*z* 166.0870 present in the MS spectra of this compound corresponds to the aromatic amino acid tyrosine (Tyr). The compound was attributed to the dipeptide Tyr-Thr, which was later decarboxylated.

The basic structure of diketopiperazine (DKP) was attributed to two of the studied metabolites, namely, **31** and **36**. Diketopiperazines are commonly biosynthesized by a diversity of organisms, which comprised mammals but also bacteria.^27^ Compound **31**, with *m*/*z* 251.1511 and a molecular formula of C_12_H_19_N_4_O_2_^+^, was found in all samples. It was identified as the diketopiperazine derived from leucine and histidine (Leu-His). The initial loss of CO in the MS spectrum^28^ supports the attribution to DKP. Additionally, the fragment *m*/*z* 178.1342 is common to peptides containing His. Ions with *m*/*z* 86.0967 and 166.0617 correspond to complementary fragments of the molecule (Supporting Information Figure G). The structure of cyclic Arg-Val was attributed to compound **36** (*m*/*z* 256.1777, C_11_H_22_N_5_O_2_^+^) (Supporting Information Figure H1). In this case, the most significant fragmentation involves the guanidine group of arginine, with the loss of CO only visible on the MS^3^ spectrum corresponding to the base peak (Supporting Information Figure H2).

The fragments *m*/*z* 145.0501 and 85.0287 on the MS^2^ spectrum of **67** (*m*/*z* 304.2130, C_15_H_30_NO_5_^+^) matched with fragments from acylcarnitine in the METLIN database.^25^ Moreover, the presence of *O*-acylcarnitines has been described in metabolomic studies on milk during bovine mastitis.^24^ Therefore, the structure of *O*-(3-hydroxy)octanoylcarnitine was attributed to this compound (Supporting Information Figure I).

Finally, a compound with *m*/*z* 249.1859, corresponding to a molecular formula of C_16_H_25_O_2_^+^ (**30**), was detected (Supporting Information Figure J). Unfortunately, the structure of this substance could not be attributed, not even tentatively. However, according to the METLIN database,^25^ fragments *m*/*z* 213.1646, 189.1640, and 135.1175 are common to prostaglandin-related compounds.

### 3.3 Evolution of Metabolite Content with Time

**Figure 2:**
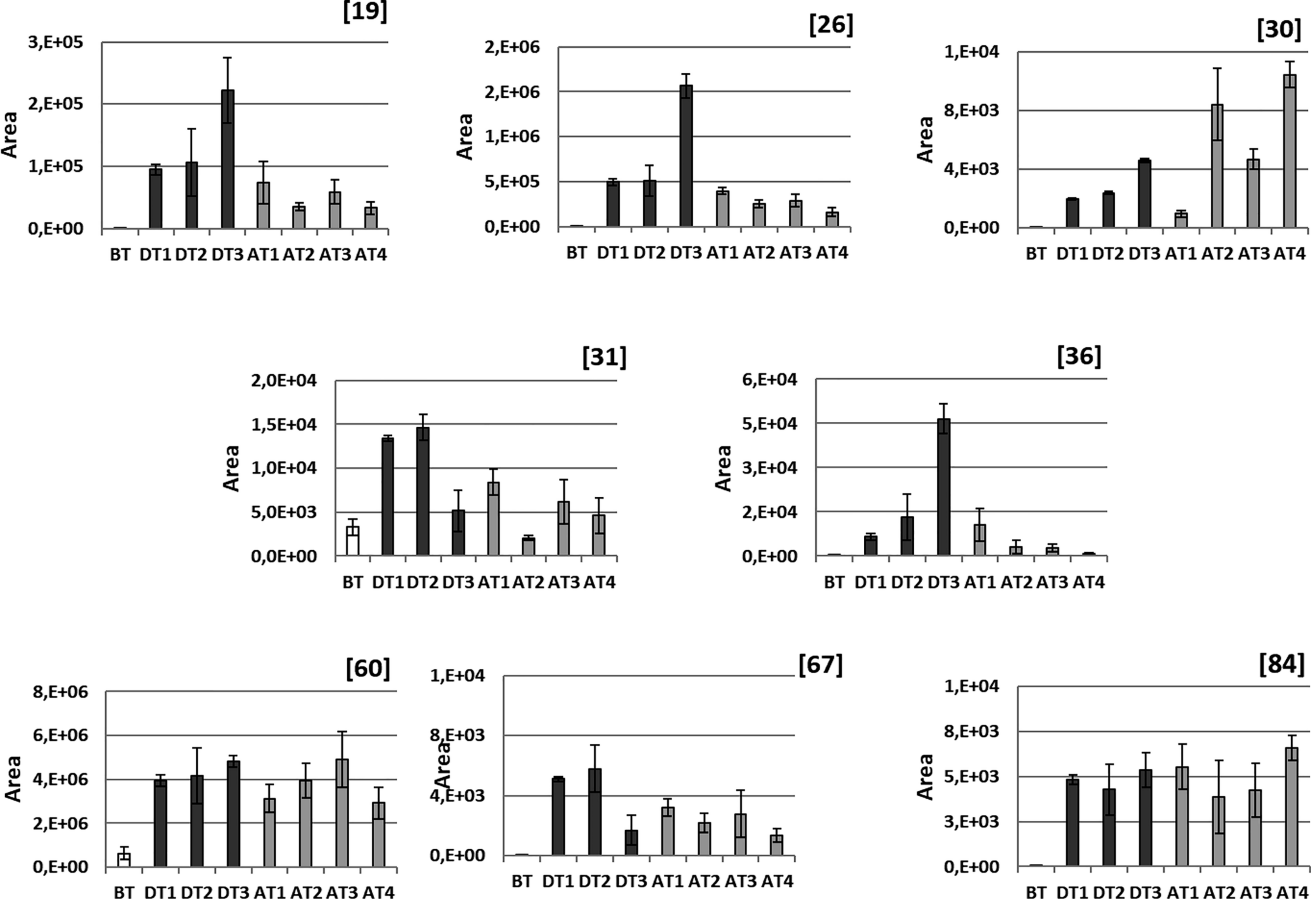
Effect of the pharmacological treatment on the behavior of the metabolites obtained. BT, white bar; DT, dark gray bar; PT, light gray bar.

To study the effect of the evolving pathology on the content of the selected compounds, their *m*/*z* areas were plotted for all nonheated samples (T0). In Figure 2, the evolution of the studied metabolites with time in which milk was obtained is shown. Compounds **31** and **60** are the only compounds monitored found in all samples, even though with a diverse intensity. Therefore, these compounds cannot be directly attributed either to the pathological process or to the pharmacological treatment, although their concentration results affected the latter once established. In contrast, the remaining monitored compounds, undetectable in BT samples, are the result of either the pathological process or the therapeutic treatment and, therefore, their evolution may be of interest in this context.

Thus, compounds **19**, **26**, **31**, **36**, and **67**, after showing a pronounced increase in concentration in DT samples, show a decrease in concentration in AT samples. This effect is particularly significant for **19**, **26**, and **36**, which experience a strong drop on the interruption of the treatment, pointing to an effect of the medication on the origin for these metabolites. Compounds **31** and **67** show also the same decreasing trend, although less pronounced. The level of these latter compounds starts lowering even during treatment (DT3), which may be a reflection of the disease remission. Alternatively, compound **60** together with compound **84** shows a sustained level after the establishment of treatment and during the monitored period. It is likely that these metabolites will also decrease in concentration with time but in a wider period than the one studied. Following the same reasoning, remission is not complete during the studied period, as it seems to indicate not only the relatively increased levels of all monitored metabolites regarding those in BT samples but also the steady increasing levels of **30**, a prostaglandin derivative, during the whole period.

### 3.4 Effect of Thermal Treatment

**Figure 3:**
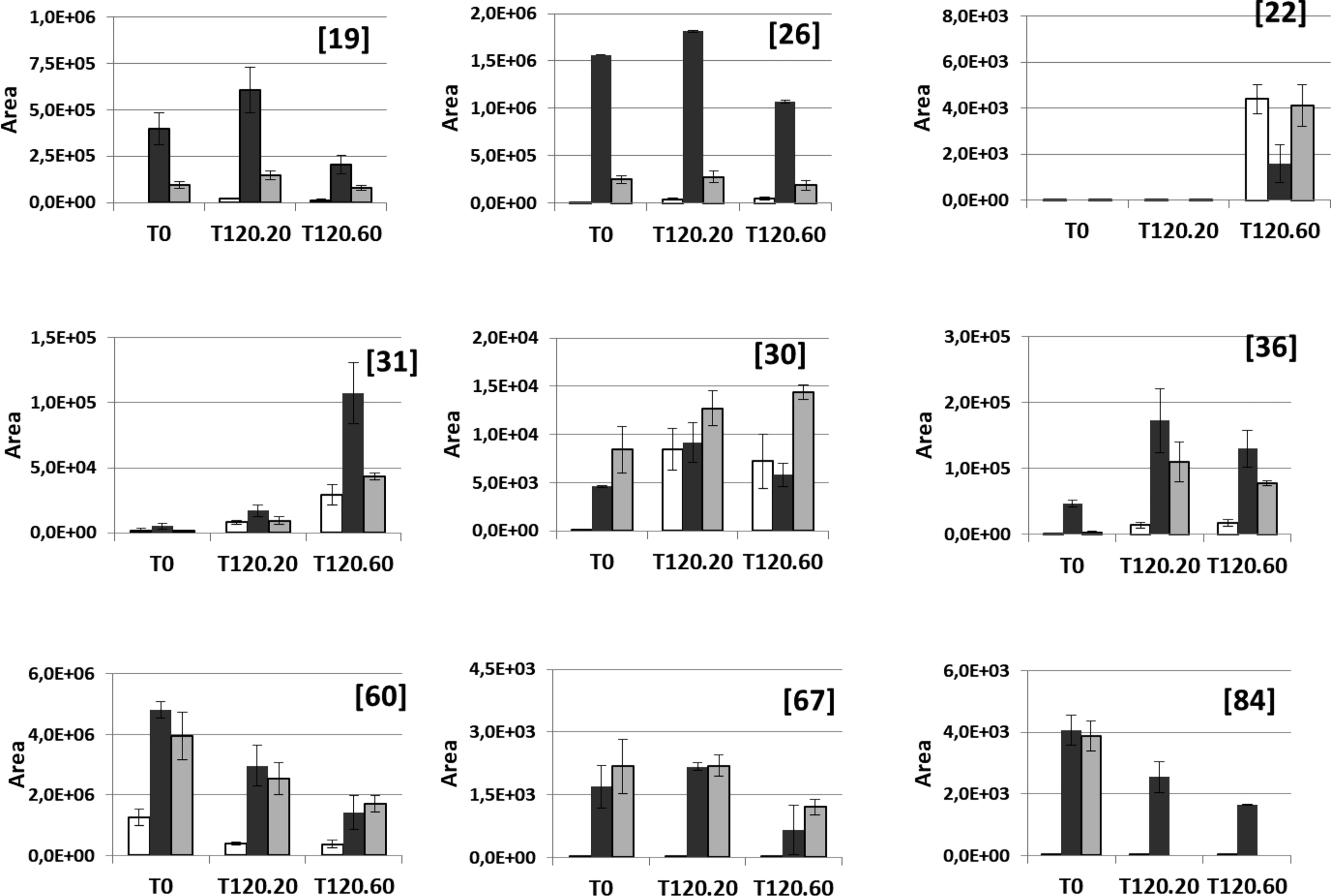
Effect of the temperature on the behavior of the metabolites obtained. BT, white bar; DT, dark gray bar; PT, light gray bar.

Temperature is usually used in milk with the aim to degrade pathogens. However, a possible effect on the degradation or increase of certain metabolites is also possible. To study the effect of temperature on the selected compounds, their *m*/*z* areas for BT, DT, and AT samples under different heating conditions (T0, T120.20, and T120.60) were considered. Figure 3 shows the dependence of the monitored compound concentration on the thermal treatment applied. For a more understandable graphic, DT3 and AT2 samples were chosen as the representative of the corresponding periods DT and AT (complete data set in the Supporting Information, Figure K).

As mentioned above, together with the destruction of pathogens, it would be expected that the temperature promotes also the degradation of diverse metabolites. This is the trend followed by compounds structurally related to ENR.^15^ However, among the compounds monitored in the present study, three different trends for the effect of temperature are observed.

First, the concentrations of **60**, **67**, and **84** clearly decrease when the sample is heated, with this effect even stronger when prolonged in time. This affects peptide derivatives (**60** and **84**) more strongly than the acyl carnitine derivative (**67**).

An opposite trend is also observed. There are compounds such as **22** and **31** whose concentrations are increased because of the thermal treatment. In the case of **22**, a decarboxylated dipeptide, it is only detected in samples subjected to prolonged heating (T120.60), which indicates the character of the thermal artifact for this compound.

Finally, although the presence of compounds such as **19**, **26**, and **36** seems to be promoted by temperature, the prolonged heating results in a degradation, which originates the increasing/decreasing profile observed.

Although quantification is not possible, it is clear that the content of the diverse compounds studied is not equivalent. There are several compounds whose area is in the order of 1000 units (**67**, **84**, and **22**), which makes their presence in samples not robust enough to consider them as biomarkers either for the pharmacological treatment or for the pathology. Even more, **22** can be considered an artifact of the intensive thermal process.

Compound **30** shows area values in the order of 10^3^ to 10^4^ continuously increasing during the studied period (Figure 2), although it is not present in BT samples. This may indicate a relationship between its content and the pharmacological treatment. Unfortunately, this compound appears after heating even in BT samples (Figure 3). Thus, **30**, a tentative prostaglandin derivative, can result from the degradation of a possible nonmonitored thermally sensitive derivative.

Other compounds, such as **19**, **26**, **31**, **36**, and **60**, show area values over 10^4^. Among them, **26** and **60** reach area values higher than 10^6^ (Figures 2 and 3). Compounds **19**, **26**, and **36** appear in samples DT and AT. They can be considered a result of the pharmacological treatment or, alternatively, the progress of the illness even if they were not detected in BT samples. Actually, Mansor^24^ proposed compound **26**, the dipeptide Leu-Leu, as a biomarker for mastitis. Compound **60**, found at a low level in BT samples, seems to maintain a considerable and steady concentration during the whole period studied and to be only slightly degraded after the thermal treatment at which samples are subjected. It has to be taken into account that the heating processes used in this study are more aggressive than those usually used in the treatment of milk. Therefore, the thermal stability and sustained high concentration of this compound make it adequate as a possible biomarker from the analytical point of view.

In conclusion, PCA studies using ion intensities as analytical data show that the score plot classifies samples as a function of temperature and pharmacological treatment. PC1 mainly described the sample behavior as a function of drug and metabolite contents, while PC2 mainly explained the influence of the thermal treatment on the compositional profiles of drug metabolites.

From the initial list of 149 compounds, whose concentrations were altered by either the pathology or the pharmacological treatment established, 60 were identified. Attention was focused on 9 of them, mainly amino acid- and peptide-related compounds.

The evolution of their content in samples as a function of pharmacological treatment time and temperature was studied. Compounds **19**, **26**, **36**, and **60** show enough persistence in time and thermal stability to be considered suitable biomarker candidates from the analytical point of view for treatment or disease.

## Supporting Information Available

Full list of compounds obtained in this work, ordered by the *m*/*z* ratio (PDF)
PCA results from the treatment of the raw data matrix with no assumptions on the nature of the most relevant biomarkers; MS spectra of compounds **19**, **22**, **26**, **30**, **31**, **36**, **60**, **67**, and **84**; effect of the pharmacological treatment on the behavior of the metabolites obtained (PDF)
