# Supplementary material for: Metabolites in Milk after Enrofloxacin Treatment and Their Persistence to Temperature
Source: J Agric Food Chem. 2022 Jul 1;70(27):8441–50. doi: 10.1021/acs.jafc.2c02230 (PMC9880995; doi:10.1021/acs.jafc.2c02230)
Supplement: Supplementary file 1 — jf2c02230_si_002.pdf [file jf2c02230_si_002.pdf]

Table A. Supplementary data. Full list of compounds obtained in this work, ordered by *m/z* ratio

	m/z	RT			*m/z*	RT			*m/z*	RT			*m/z*	RT			*m/z*	RT
1	200,1761	2,2		31	251,1506	2,1		61	296,1692	3,7		91	350,1727	4,8		121	387,2023	7,0
2	201,1176	7,4		32	255,1365	2,4		62	302,1386	3,4		92	351,1981	5,3		122	388,2098	5,7
3	204,1356	2,2		33	256,1739	2,4		63	302,1386	4,1		93	352,1849	2,5		123	391,1730	4,2
4	205,0696	4,6		34	256,1739	3,2		64	302,1386	4,5		94	352,1849	5,1		124	393,2092	3,6
5	212,1710	7,2		35	256,1739	5,7		65	302,1386	5,7		95	353,1185	6,5		125	397,1901	2,2
6	213,0561	3,7		36	256,1739	3,2		66	304,1312	4,5		96	356,2010	3,4		126	398,2372	7,1
7	215,1341	2,8		37	257,1466	4,1		67	304,2120	4,0		97	358,2133	5,9		127	399,2401	3,2
8	215,1405	3,9		38	258,1821	5,8		68	305,1606	6,6		98	359,1259	5,6		128	400,2050	6,1
9	217,1427	6,8		39	261,1326	3,5		69	306,1226	2,3		99	360,1389	6,9		129	403,0633	5,1
10	217,1427	7,7		40	262,1313	5,5		70	306,1281	3,6		100	360,1721	5,7		130	404,2133	6,3
11	218,1282	2,3		41	263,0844	6,9		71	307,1242	2,5		101	360,1953	2,5		131	405,2109	3,4
12	219,1293	5,7		42	264,1636	6,8		72	311,2029	2,3		102	362,2285	2,2		132	405,2109	7,0
13	228,1695	2,2		43	264,1702	2,7		73	313,0661	2,3		103	365,2050	2,6		133	406,2219	2,8
14	228,1786	5,7		44	265,1560	2,1		74	314,1322	5,0		104	365,2050	4,4		134	406,2219	3,3
15	228,1786	3,1		45	265,1560	3,1		75	317,1570	8,4		105	366,2035	2,8		135	406,2331	3,5
16	229,1206	3,6		46	265,1560	4,1		76	322,1813	3,0		106	367,1843	2,3		136	408,1637	2,2
17	229,1820	8,4		47	266,1598	2,2		77	329,1517	3,5		107	369,2364	2,3		137	410,2321	7,0
18	231,1717	2,3		48	266,1598	4,1		78	329,1517	5,1		108	369,2364	3,0		138	412,2247	6,6
19	231,1717	3,0		49	266,1598	5,4		79	330,1650	4,2		109	369,2364	3,5		139	419,2263	5,5
20	231,1717	5,7		50	269,1742	8,4		80	332,1425	5,1		110	371,2300	2,9		140	421,2179	4,2
21	233,0933	6,3		51	275,1081	4,6		81	332,1826	8,4		111	373,1694	5,1		141	424,2325	6,8
22	239,1375	7,7		52	278,1800	7,3		82	334,1219	6,4		112	373,2443	2,5		142	425,2328	3,5
23	242,1757	5,6		53	287,1159	5,7		83	334,1537	5,0		113	376,1705	4,5		143	426,2078	5,2
24	242,1757	5,8		54	288,1160	2,6		84	334,1795	5,8		114	376,1925	3,6		144	427,1703	3,1
25	245,1871	3,4		55	288,1441	2,3		85	335,1078	7,7		115	376,2163	5,5		145	427,1756	3,2
26	245,1871	5,1		56	288,1441	2,9		86	338,1675	5,1		116	377,1516	4,0		146	429,2263	5,5
27	247,1834	3,9		57	288,1441	4,2		87	345,1893	4,2		117	378,1734	5,8		147	433,2475	4,3
28	247,1914	8,4		58	288,1441	5,1		88	345,2141	2,2		118	379,1694	4,0		148	434,2384	7,5
29	249,1637	3,7		59	289,1803	3,2		89	346,1216	6,5		119	379,2133	3,8		149	443,2386	8,5
