# Supplementary material for: Metabolites in Milk after Enrofloxacin Treatment and Their Persistence to Temperature
Source: J Agric Food Chem. 2022 Jul 1;70(27):8441–50. doi: 10.1021/acs.jafc.2c02230 (PMC9880995; doi:10.1021/acs.jafc.2c02230)
Supplement: Supplementary file 2 — jf2c02230_si_003.pdf [file jf2c02230_si_003.pdf]

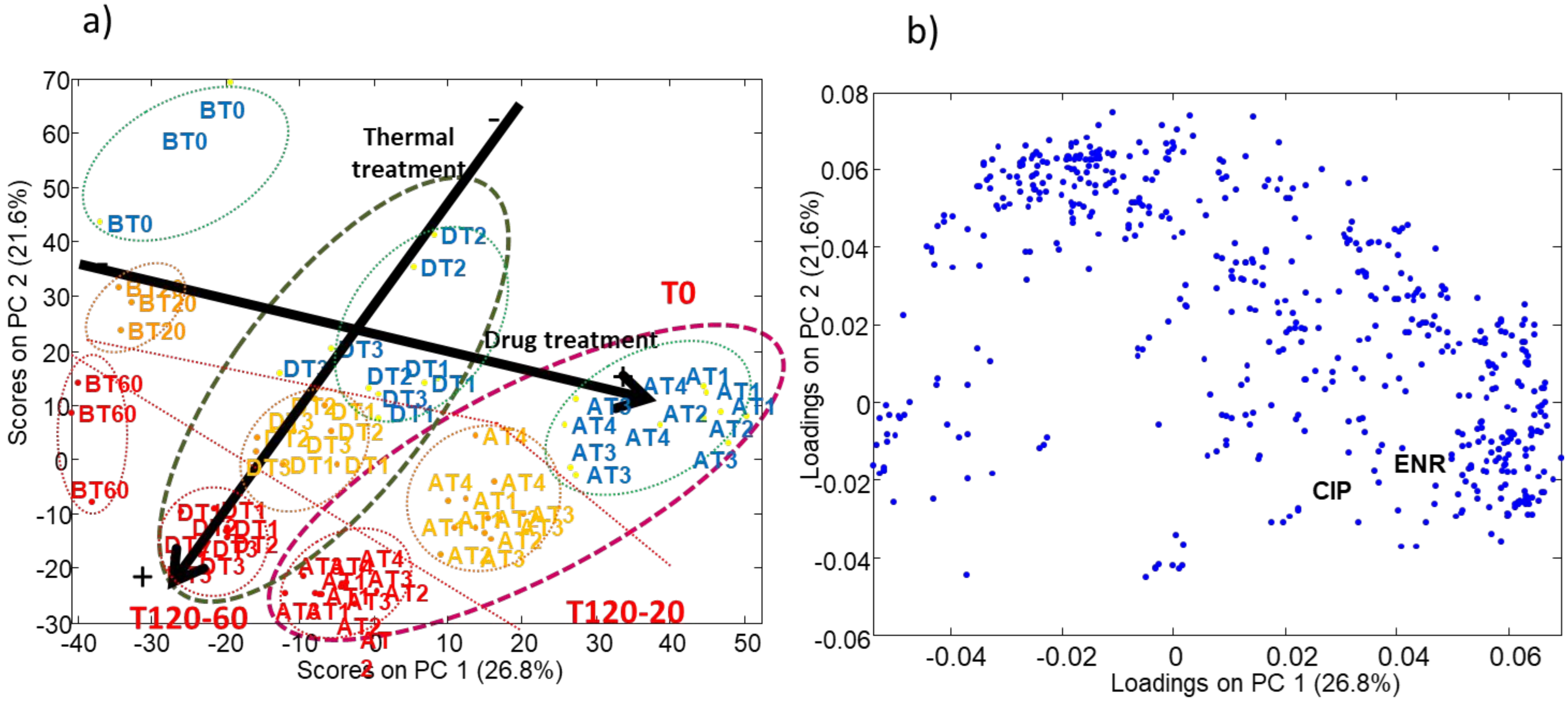

Figure A. PCA results from the treatment of the raw data matrix with no assumptions on the nature of the most relevant biomarkers. (a) plot of scores; (b) plot of loadings.

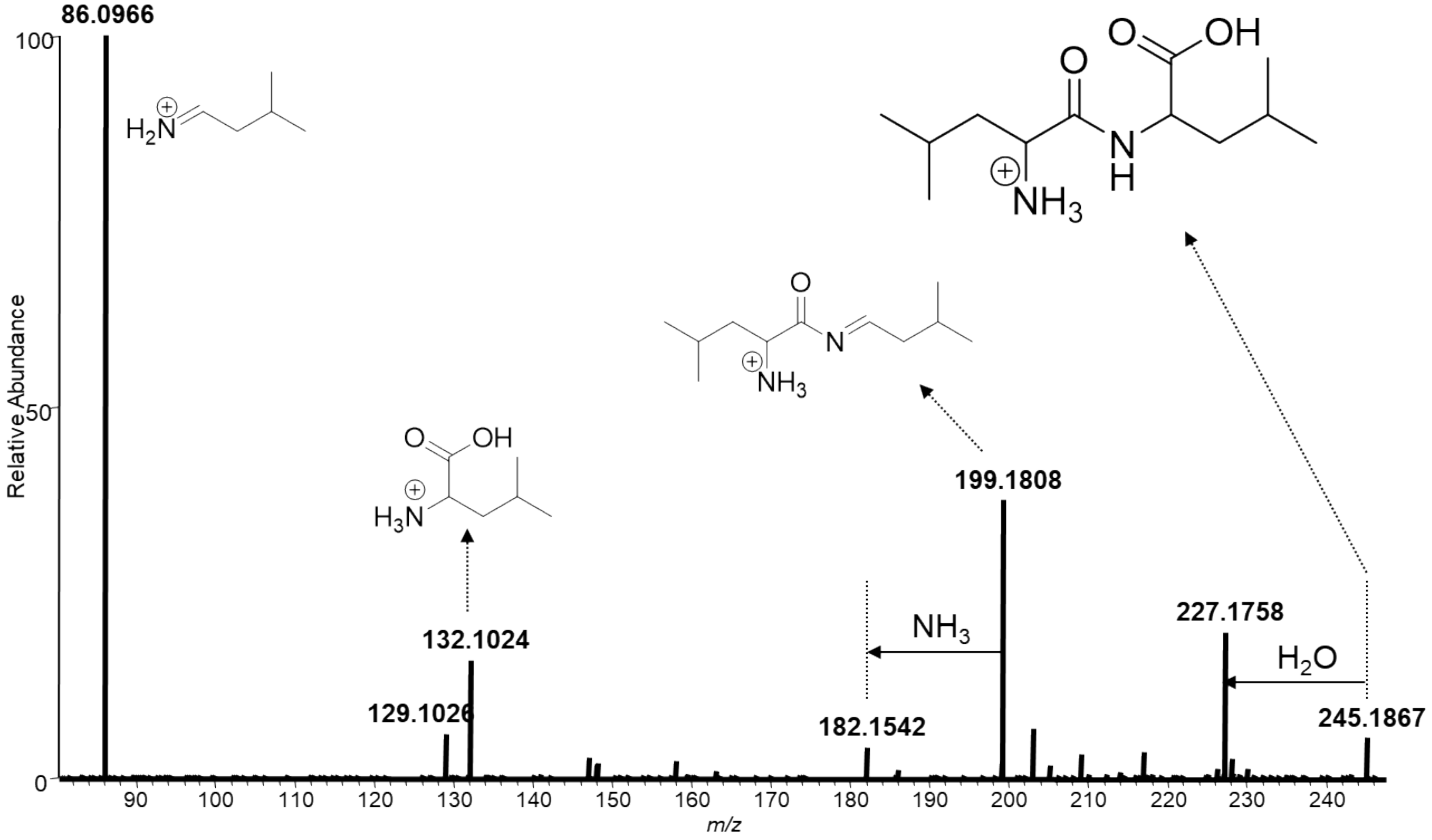

Figure B, compound **26**

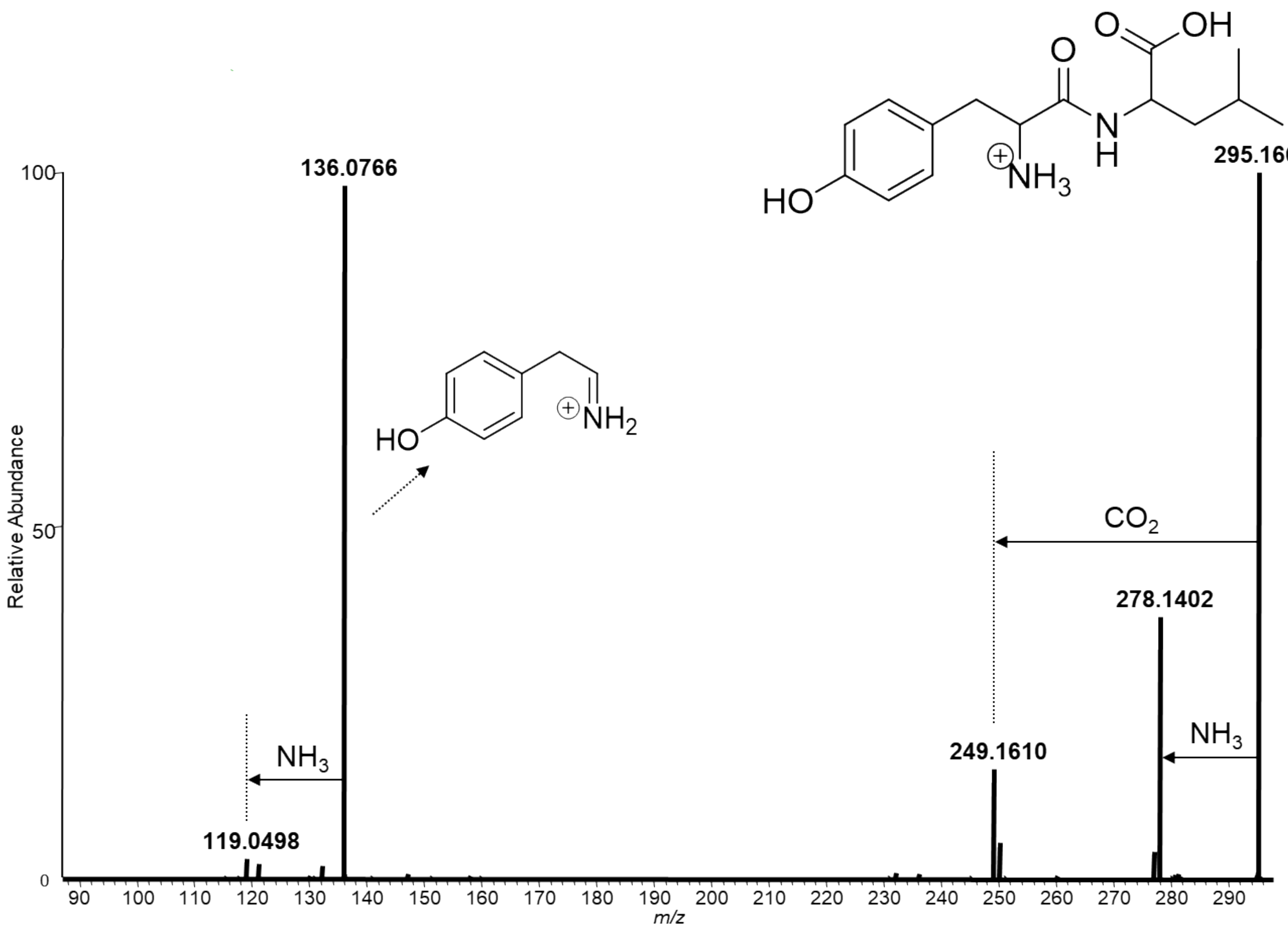

Figure C, compound **60**

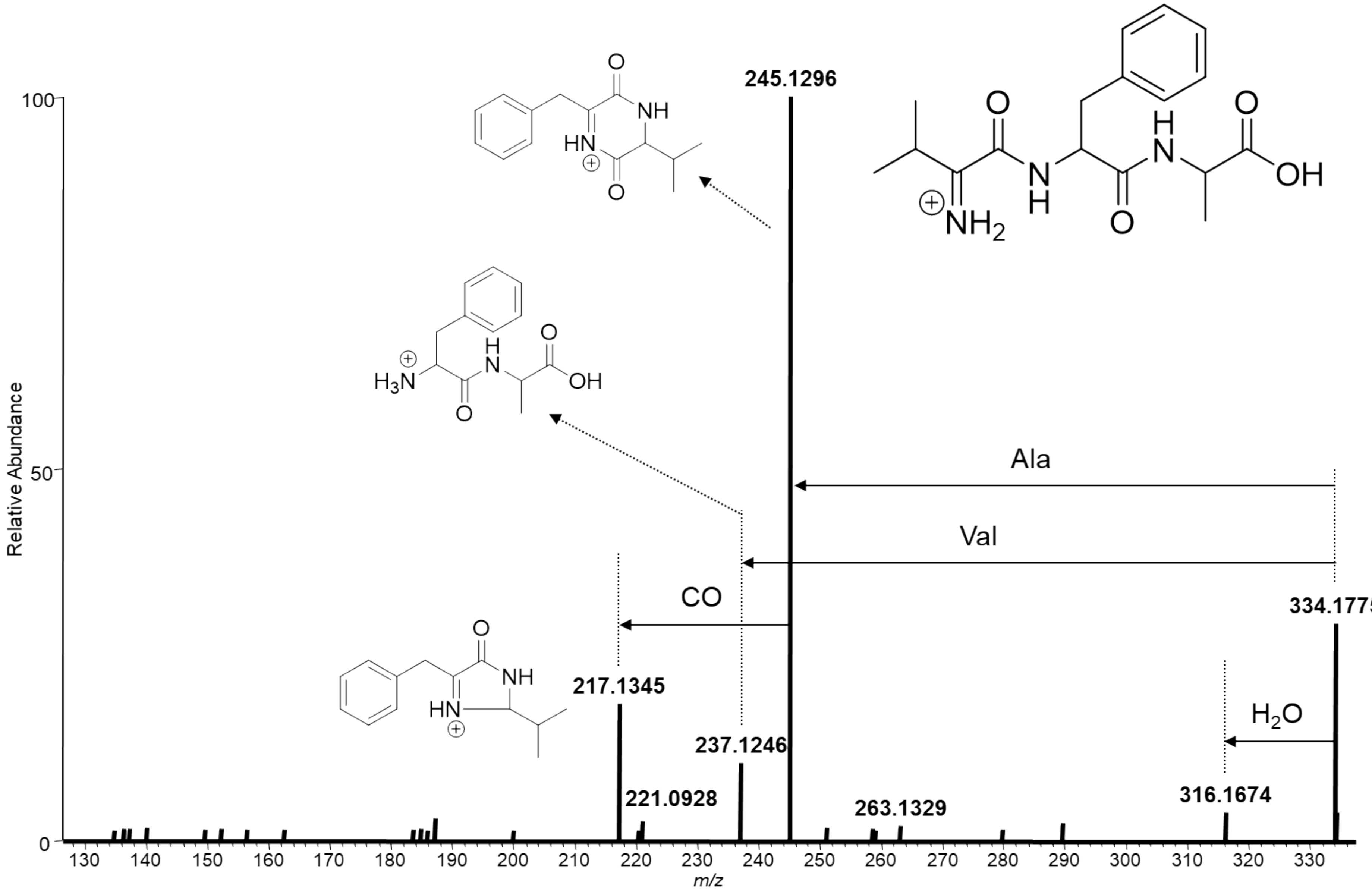

Figure D, compound **84**

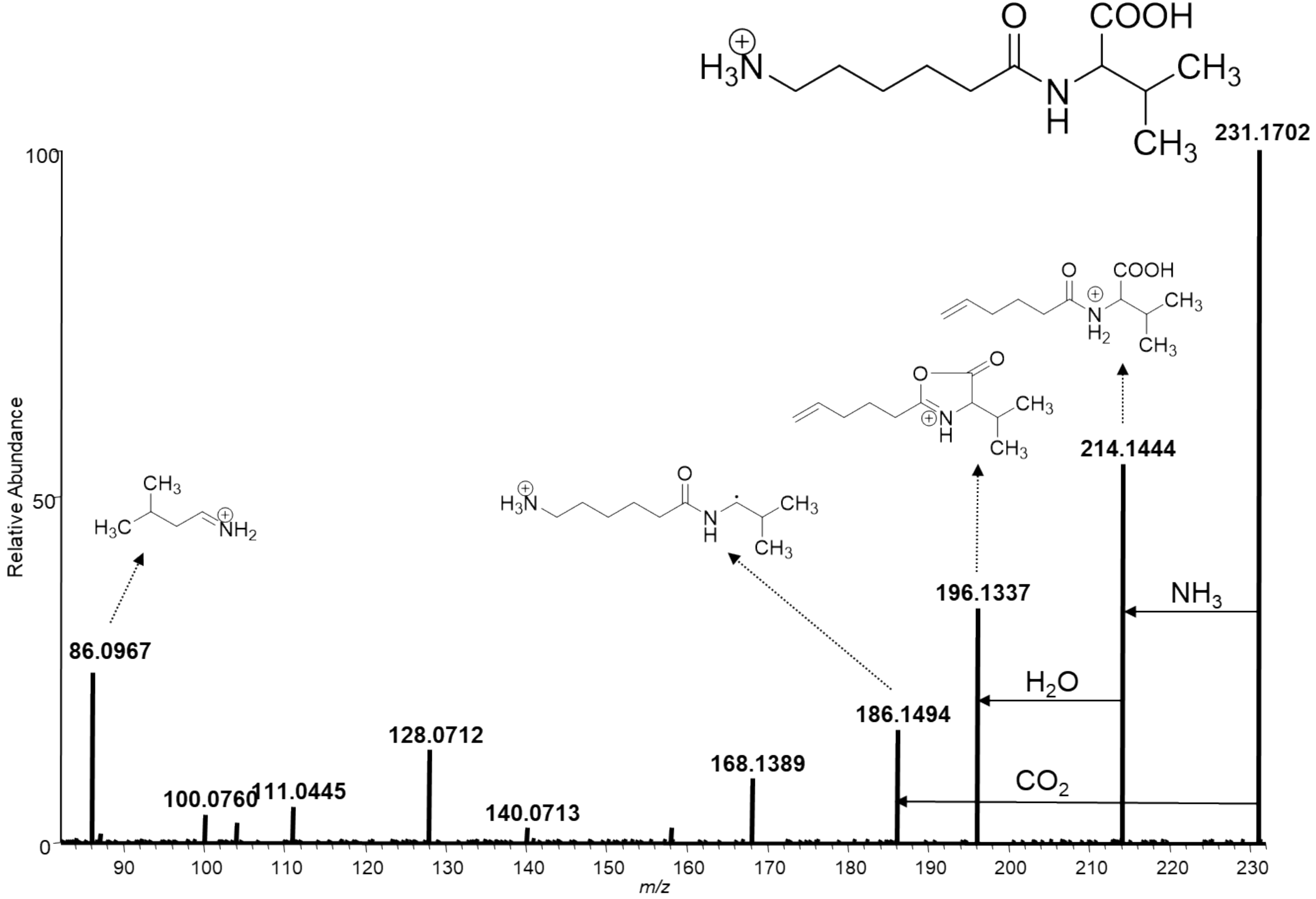

Figure E, compound **19**

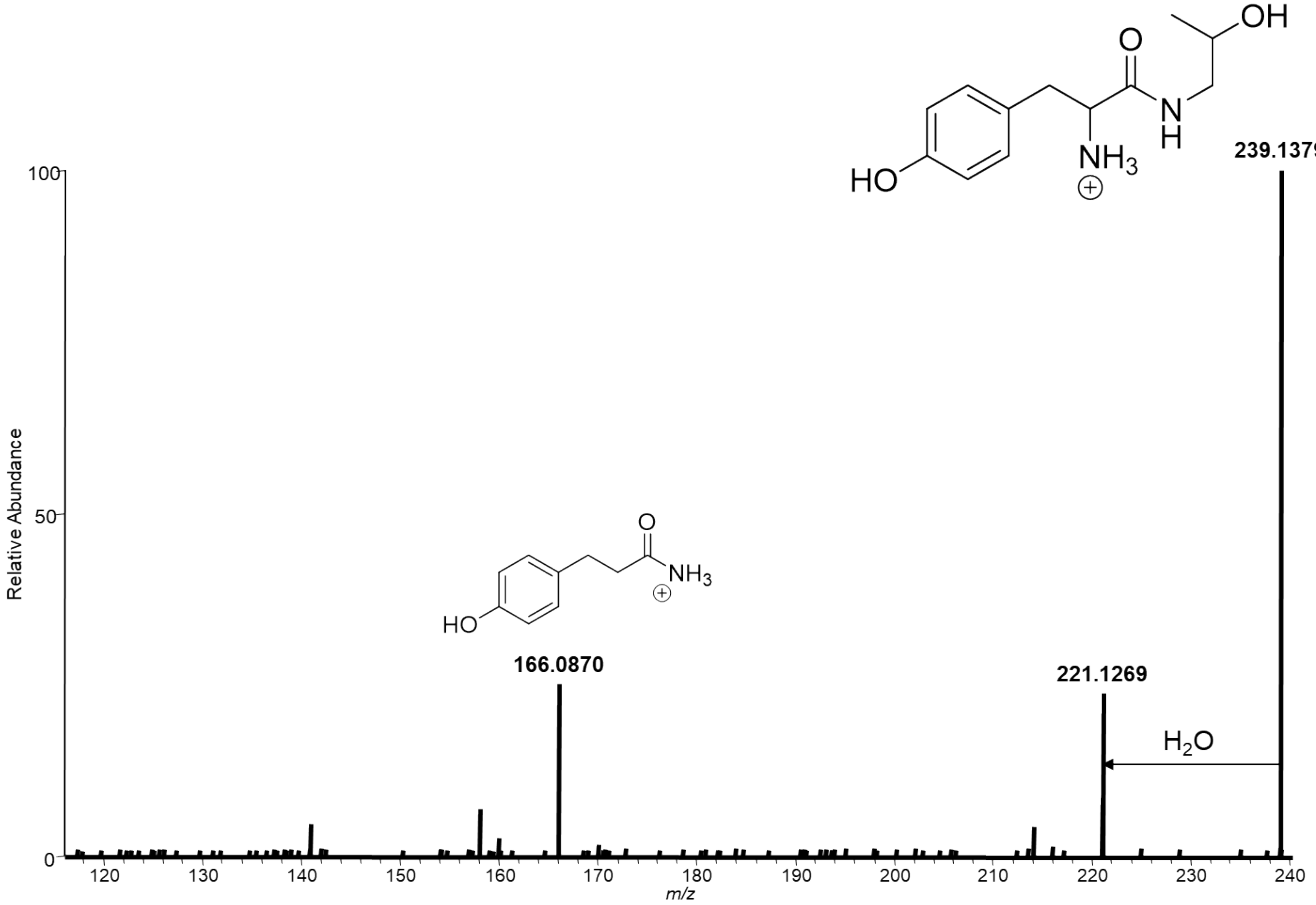

Figure F, compound **22**

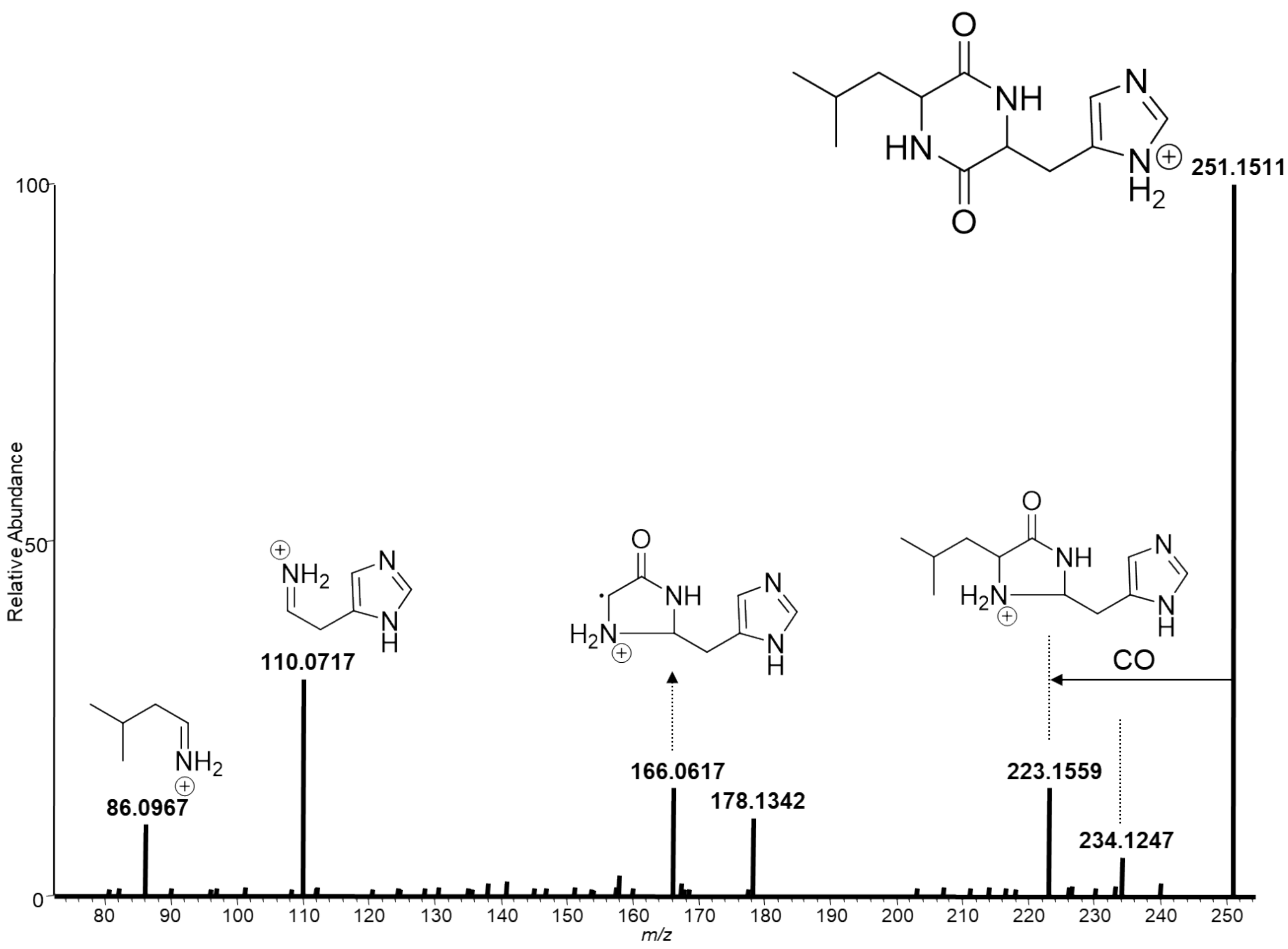

Figure G, compound **31**

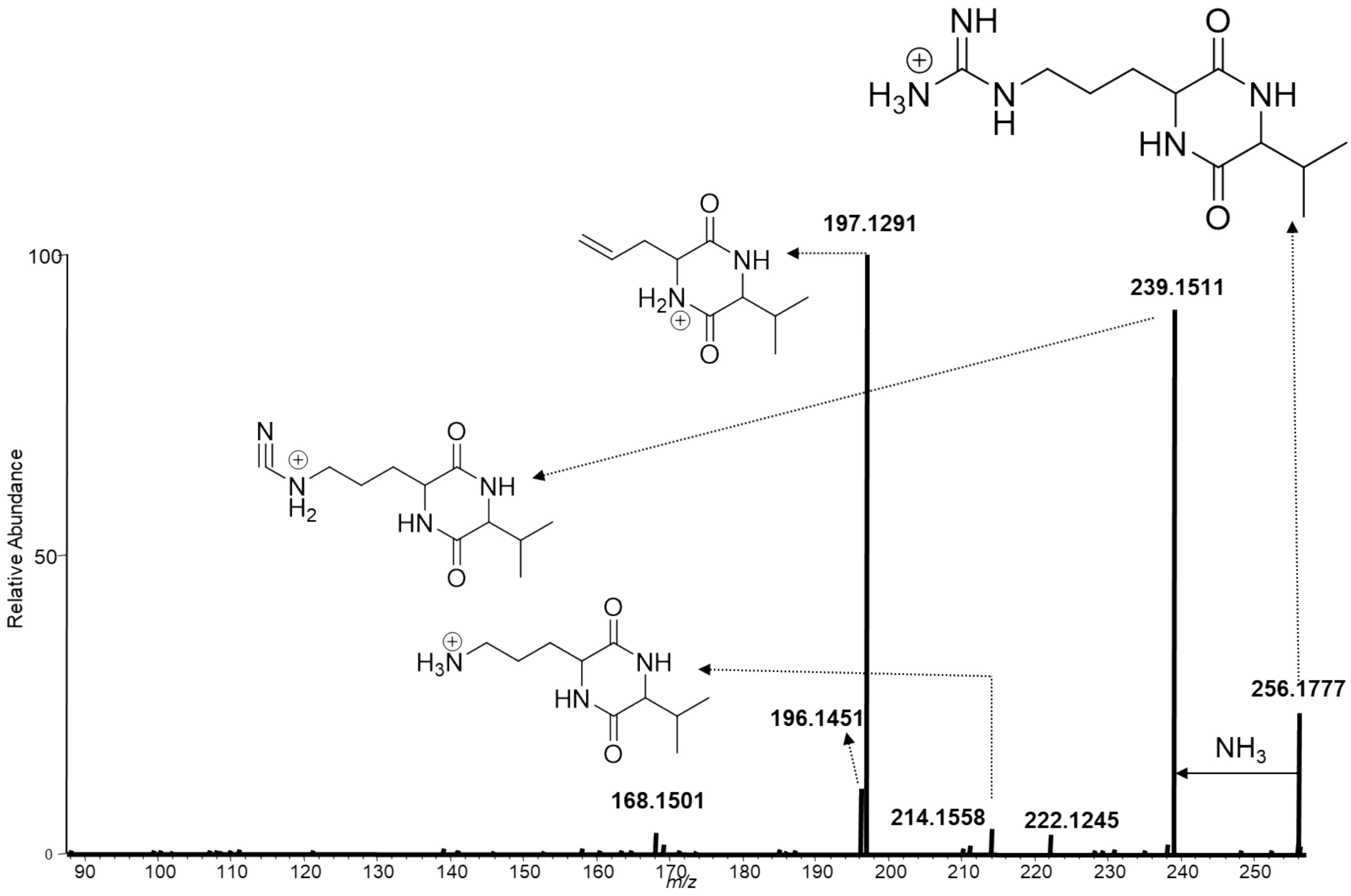

Figure H1, compound **36**

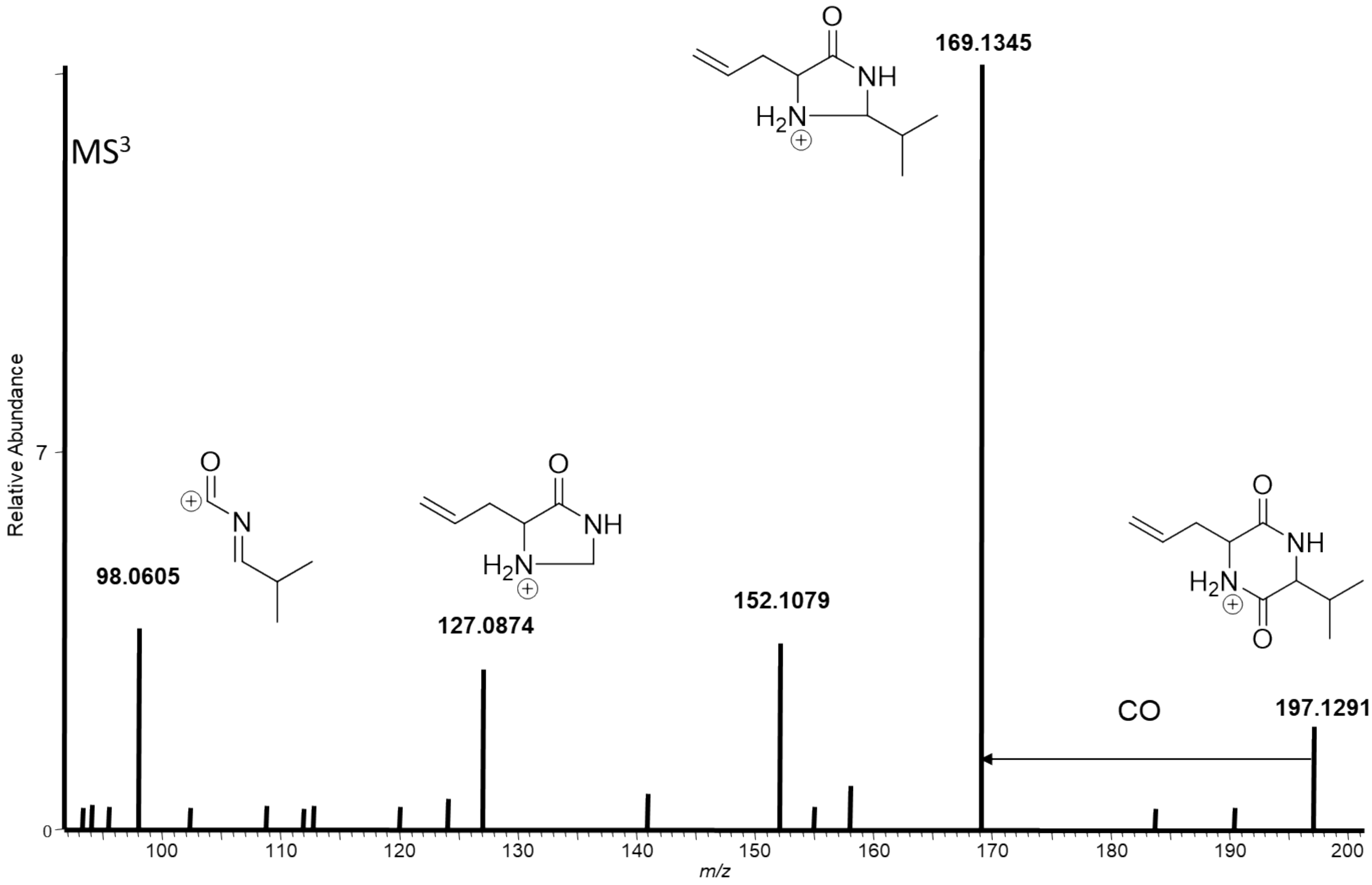

Figure H2, compound **36**

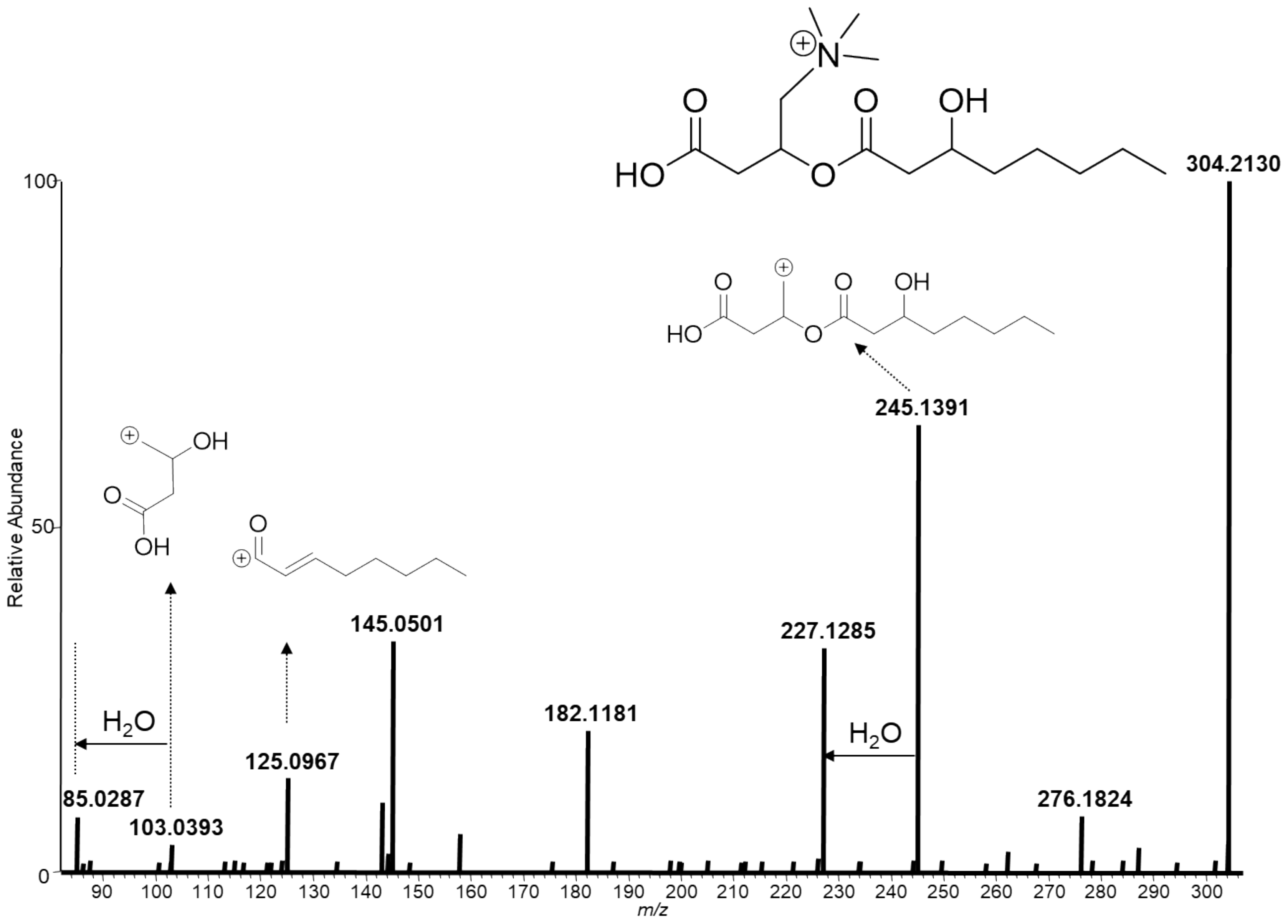

Figure I, compound **67**

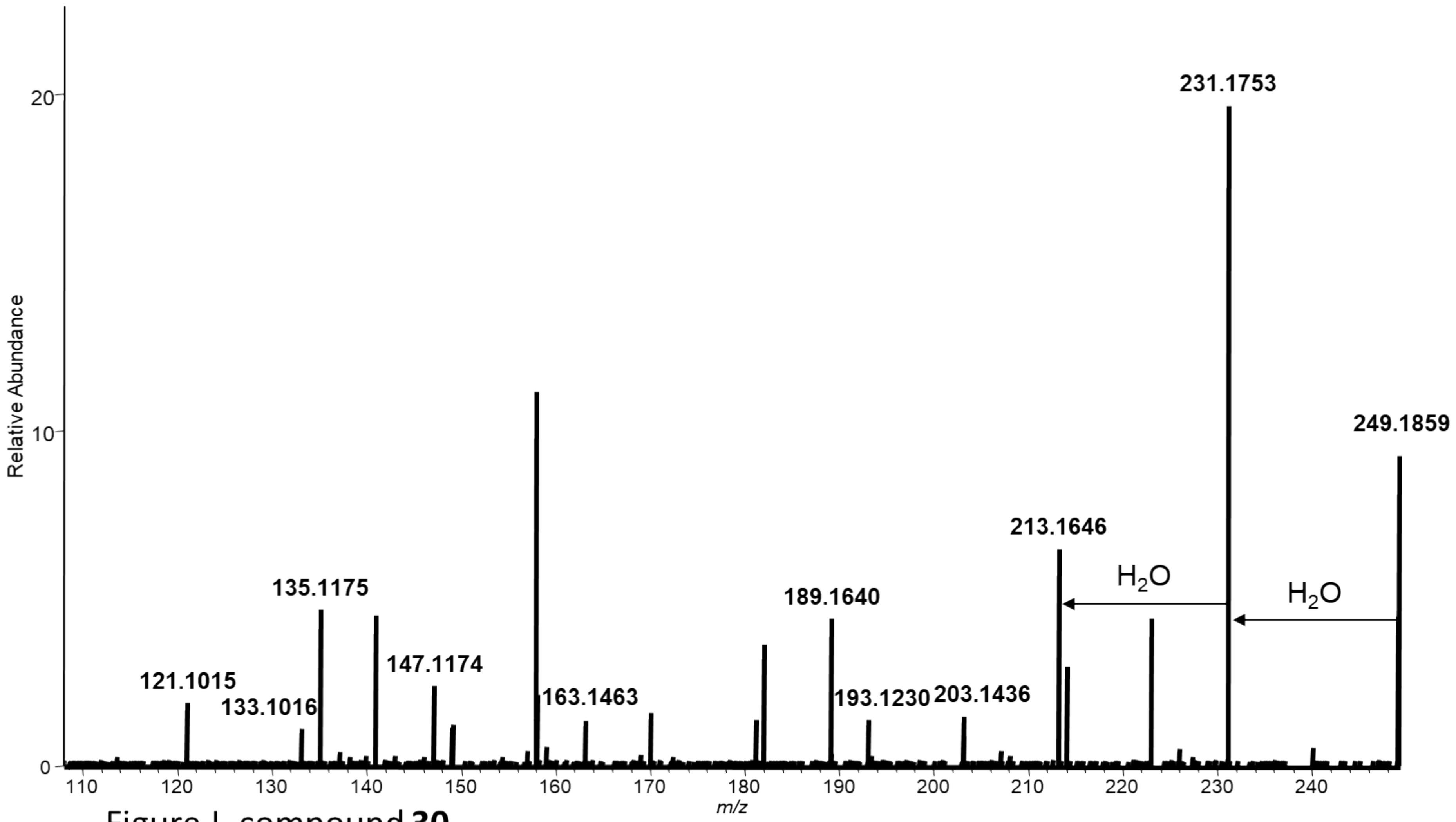

Figure J, compound **30**

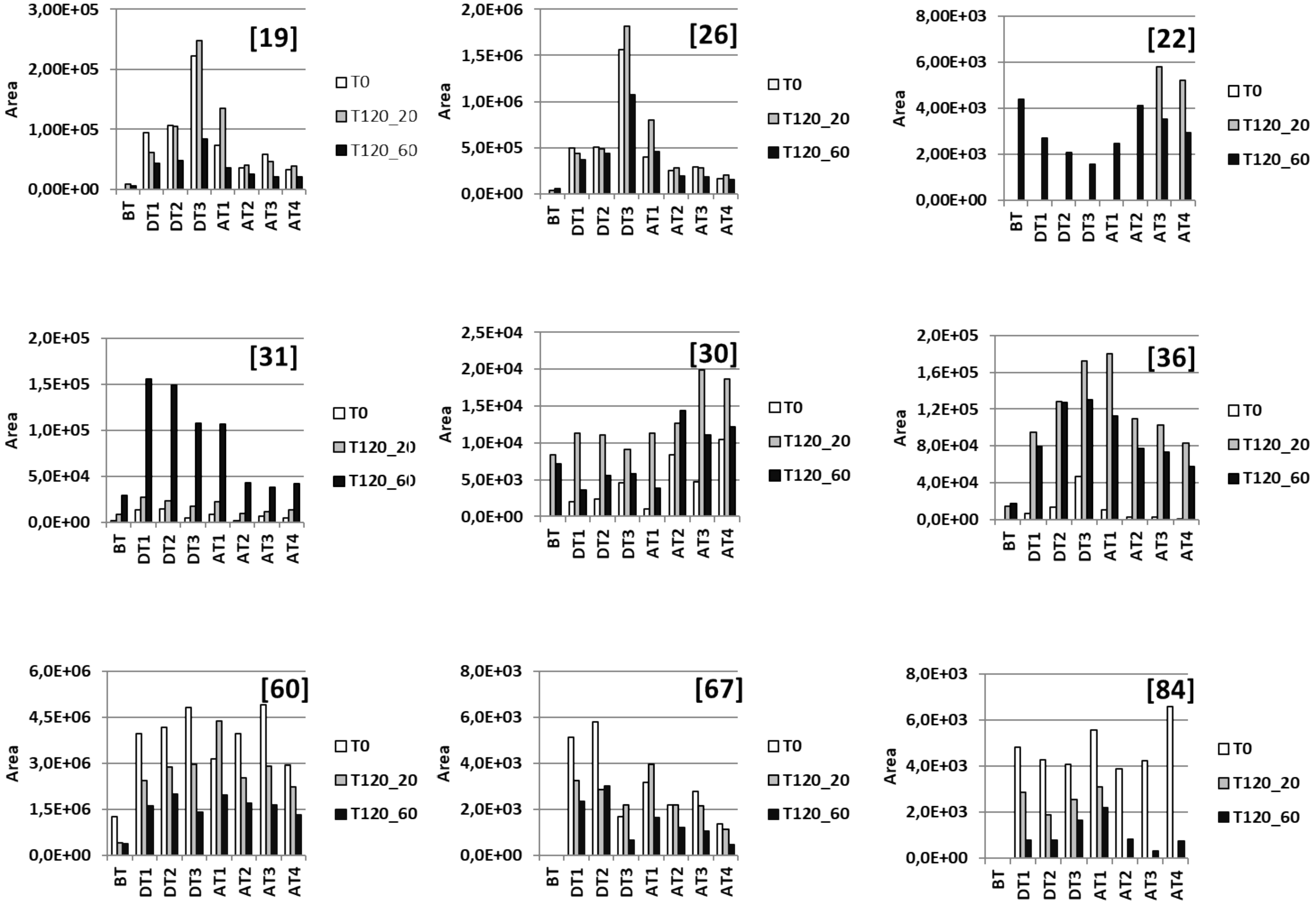
[19]
[26]
[22]
[31]
[30]
[36]
[60]
[67]
[84]
Area
T0
T120_20
T120_60
BT
DT1
DT2
DT3
AT1
AT2
AT3
AT4
